# Enhanced osteogenesis and antibacterial activity of bioactive glass-coated, epsilon-poly-L-lysine-loaded, polycaprolactone 3D-printed scaffolds with potential use in bone tissue engineering

**DOI:** 10.1007/s10856-026-07065-8

**Published:** 2026-05-05

**Authors:** Eddy Shan, Liang Feng, Elena Figuero, Mariano Sanz, Maurizio Tonetti, Jinwu Wang

**Affiliations:** 1 Shanghai key Laboratory of Stomatology, Department of Oral Implantology, Shanghai Perio-Implant Innovation Center, Shanghai Ninth People Hospital, College of Stomatology, National Center of Stomatology, National Clinical Research Center for Oral Diseases, Shanghai Jiao Tong University School of Medicine, Shanghai Jiao Tong University, Shanghai, China; 2 Etiology and Therapy of Periodontal and Peri-implant Diseases (ETEP) Research group, Complutense University of Madrid, Madrid, Spain; 3 Shanghai Key Laboratory of Orthopedic Implants, Department of Orthopedic Surgery, Shanghai Ninth People’s Hospital, Shanghai Jiao Tong University School of Medicine, Shanghai, China

## Abstract

This study evaluated the structural properties and biological in vitro performance of 3D-printed polycaprolactone (PCL) scaffolds, coated with bioactive glass (BG) and functionalized with epsilon-poly-L-lysine (EPL) when co-cultured with rat bone marrow mesenchymal stem cells. The physicochemical characterization included scanning electron microscopy, energy dispersive spectroscopy, x-ray photoelectron spectroscopy, Fourier transform infrared spectroscopy and degradation kinetics. The biological characterization assessed antibacterial activity using a disk diffusion assay, cell viability of rat bone marrow mesenchymal stem cells with confocal laser scanning microscopy, cell proliferation and cytotoxicity with tetrazolium assay and relative gene and protein expression through reverse transcriptase quantitative polymerase chain reaction and western blot. The physicochemical characterization showed a successful and homogeneous distribution of the ceramic component and confirmed EPL-loading. The PCL-BG-EPL scaffolds demonstrated antibacterial activity against *Staphylocccus aureus* for up to 7 days. Cell proliferation was significantly higher for PCL-BG and PCL-BG-EPL scaffolds, but cytotoxicity also increased in both groups when comparing day 3 and day 7. The osteogenic potential was enhanced for BG-coated scaffolds, with an upregulation of *Alpl, Opn*, and *Col1a1* genes when compared to controls. However, no significant differences were detected for protein synthesis among the three groups. Composite scaffolds exhibited favorable technical properties and superior biological performance over pure PCL scaffolds.

**Figure:**
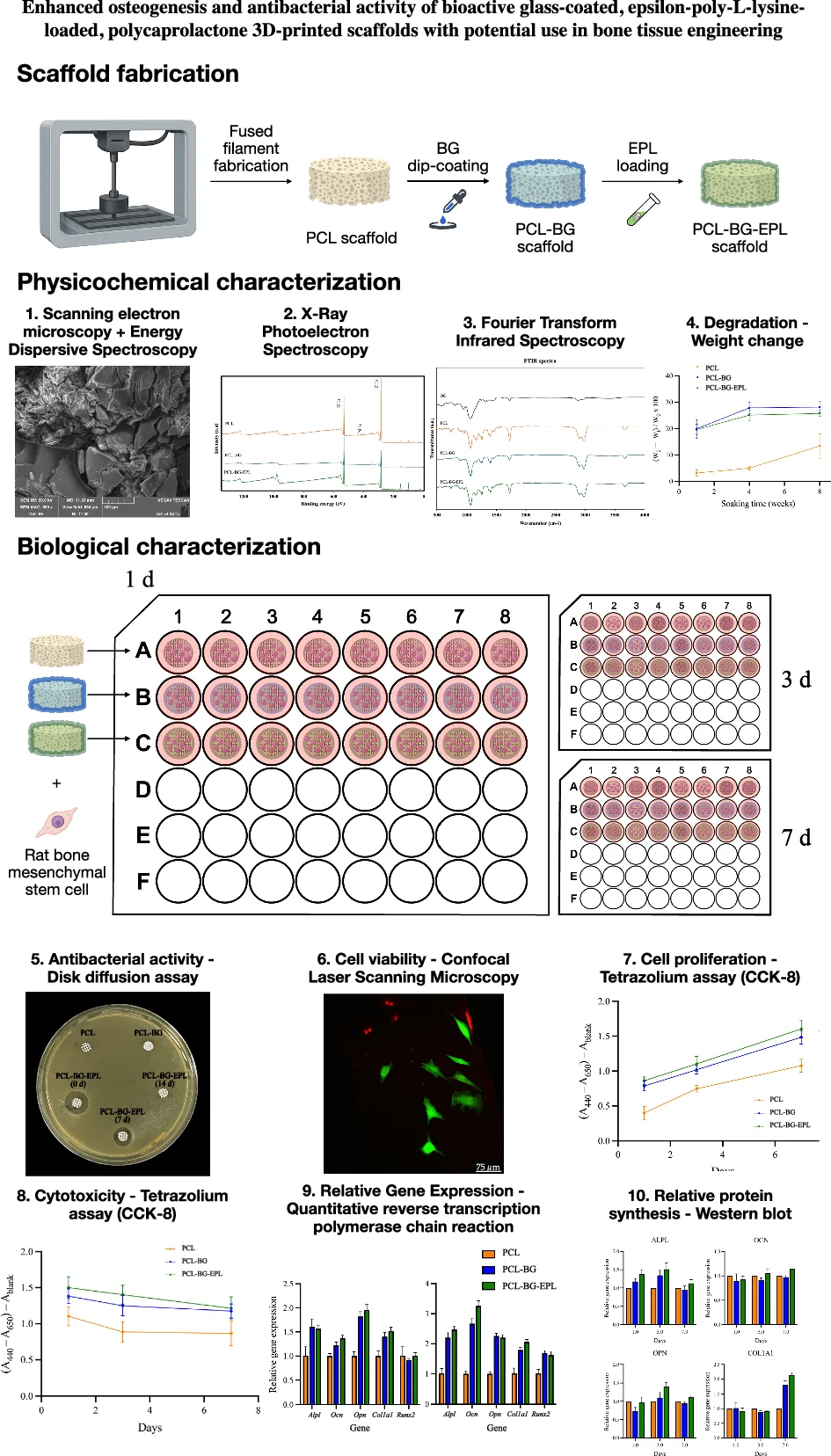

## Introduction

Tooth loss triggers a series of physiologic remodeling processes that can ultimately lead to alveolar ridge atrophies [1, 2]. In the context of tooth replacement with implant therapy, this remodeling can lead to unfavorable scenarios where soft and hard tissue augmentations may be necessary before or simultaneously with implant placement [3]. Bone tissue engineering aims to reconstruct hard tissue loss through a combination of innovative biomaterials, cell sources, and bioactive molecules. Along with the current trend of avoiding bone replacement grafts, autogenic and animal-derived sources are being replaced with synthetic scaffolding materials as promising tools in tissue engineering [4, 5].

The development of synthetic scaffolds enables the manufacturing of 3D-printed, highly porous structures [6]. These interconnected macroporous networks serve as tissue matrices that promote cell adhesion and proliferation, nutrient delivery, and vascularization [7, 8]. Various materials have been tested for scaffold manufacturing, including synthetic biodegradable polymers, such as polycaprolactone (PCL), which are the most widely studied materials, showing adequate biocompatibility and mechanical properties [9–11]. However, biodegradable polymers alone possess an inherent low hydrophilicity, which limits their ability to interact effectively with biological systems and decreases their bioactivity and osteoconductivity [12, 13]. Consequently, their performance in promoting bone regeneration and integration with the surrounding tissues may be limited [9]. To improve these qualities, composite materials are often incorporated to enhance their interaction with the biological environment, hence boosting their effectiveness in tissue engineering [14, 15].

Since first being discovered by Hench et al. in 1969 [16], bioactive glasses have been proposed to enhance the biological performance of composite scaffolds due to their bioactivity, ion releasing ability and mechanical properties, making them ideal candidates for applications in bone tissue regeneration [17, 18]. It has been shown that the implantation of these materials leads to the formation of a bonelike calcium phosphate (CaP) layer on the biomaterial, allowing it to bond with living bone [19, 20].

However, bone grafting procedures in the maxillofacial area remain subject to different degrees of complications, including those of infectious nature [21]. Different strategies are being implemented to prevent infection risks, such as local antibiotic delivery, among which antibiotic-impregnation of bone grafts has been explored [22]. Vancomycin-loaded polylactic acid (PLA) - mesoporous BG scaffolds have demonstrated sustained release and antibacterial activity against *Staphylococcus aureus* without compromising cytocompatibility [23]. But since antimicrobial resistance has become one of the main public health challenges today, natural compounds, antimicrobial peptides, and phage therapies are emerging as alternative antimicrobial strategies [24, 25]. Epsilon-poly-L-lysine (EPL) is a natural cationic homo-poly-amino acid that exhibits highly effective broad-spectrum antibacterial activity and remains stable under both acidic and alkaline conditions. Additionally, it is non-toxic, water-soluble, and biodegradable [26, 27]. These properties make EPL a viable alternative to traditional antibiotics and could be utilized in conjunction with scaffolds that have an intrinsic antimicrobial effect, thereby preventing postsurgical bone infections [28]. The incorporation of the cationic EPL molecule can be performed through dipping, where the EPL bonds with negatively charged surfaces like anionic polymers through electrostatic interactions and covalent bonds [29].

In the manufacturing of these types of composite scaffolds, 3D printing is considered a validated tool to develop customized solutions for bone regeneration [9]. Fused deposition modeling (FDM) uses filament extrusion to create 3D components based on computer-aided design models [30, 31]. These features may make 3D-printed PCL-BG scaffolds ideal alternatives to traditional guided bone regeneration biomaterials, as they enhance clinical handling, improve dimensional stability, and reduce the risk of infections after bone augmentation procedures when doped with EPL.

This research aimed to determine the physicochemical properties and biological performance of PCL-BG, EPL-loaded scaffolds co-cultured with rat bone marrow mesenchymal stem cells (rBMSCs), in terms of surface topography, elemental composition, antibacterial capacity, cell viability, cell proliferation, cytotoxicity, osteogenic gene expression, and protein synthesis.

## Materials and methods

### Study design and experimental groups

The in vitro characterization included eight assays evaluating the technical properties, osteogenic potential, and antibacterial capacity of three scaffold groups: two experimental groups – PCL scaffolds coated with BG, either with (PCL-BG-EPL) or without (PCL-BG) EPL loading – and a control group of pure PCL scaffolds.

Biological assays were conducted with 3 to 9 replicates (the range reflects the optimization of the sample size, with more variable assays requiring higher replicate numbers). In all experiments, scaffolds were seeded with rBMSC cells and harvested at 1, 3, and 7 days for endpoint analysis.

### Scaffold design and fabrication

Polycaprolactone scaffolds were produced using FDM with an extruding 3D printer (SK1, TwoTrees Technology Co., Shenzhen, China), employing a 1.75 mm diameter PCL filament (RESOMER® Filament C D1.75, Evonik Operations GmbH, Kirschenallee, Germany) and a 0.4 mm nozzle, with an extrusion temperature of 130 °C and 10 mm/s printing speed. The scaffolds were designed with a cylindrical shape, measuring 5 mm in diameter and 3 mm in height.

This report describes a composite scaffold made with photocurable BG coated onto the macroporous framework of PCL scaffolds through a dip-coating method. The BG precursor was obtained by mixing a nonionic block copolymer F-127 (Pluronic® F-127, 53 g/L, CAS No. 9003-11-6, BASF Corporation, Germany) as a surfactant, together with tetraethyl orthosilicate (TEOS, 0.304 mol/L, CAS No. 78-10-4, Reagent Chemical, Guangdong, China); 3-(trimethoxysilyl)propyl methacrylate (0.130 mol/L, CAS No. 2530-85-0, Aladdin Scientific, Shanghai, China); calcium nitrate tetrahydrate (Ca(NO₃)₂·4H₂O, 0.053 mol/L, CAS No. 13477-34-4, Reagent Chemical, Guangdong, China); triethyl phosphate (TEP, 0.022 mol/L, CAS No. 78-40-0, Reagent Chemical, Guangdong, China) [32, 33]. All components were added in methanol (CAS No. 67-56-1, Reagent Chemical, Guangdong, China) with 6 mL HCl (0.5 mol/L, CAS No. 7647-01-0, Reagent Chemical, Guangdong, China). The mixture was stirred overnight, and the methanol was subsequently removed by rotary evaporation. A photoinitiator (lithium phenyl-2,4,6-trimethylbenzoylphosphinate, CAS No. 85073-19-4, Yuanye Bio-Technology Co., Shanghai, China) was added to the BG precursor at a 0.25% concentration.

For the BG-coating, the PCL scaffolds were dipped into the BG precursor sol-gel and photocured using a light source wavelength of 405 nm, with a 10 mW/cm^2^ intensity for 1 min. The PCL-BG scaffolds were washed with distilled water ten times and immersed overnight to remove copolymer F-127 and photoinitiator residues, allowing methanol to evaporate. Functionalziation was performed by dissolving EPL hydrochloride (Shanghai Macklin Biochemical Technology, Shanghai, China) in distilled water at a concentration of 10 mg/mL. The scaffolds were then immersed in 50 mL EPL solution with gentle shaking for 24 h at room temperature.

The scaffolds were kept at 4 °C. Before seeding cells, all scaffolds were sterilized with UV radiation (30 minutes on each side).

### Cell culture

Rat bone marrow mesenchymal stem cells isolated from the marrow of the femoral head of male, 4-week-old, Sprague-Dawley rats (Shengchang Biotechnology, Shanghai, China) were used as primary cells. The cells were cultured in 100 mm cell culture dishes (LabSelect, Anhui, China) using Dulbecco’s Modified Eagle Medium (DMEM, Gibco, Thermo Fisher Scientific, Waltham, Massachusetts, USA), supplemented with 10% fetal bovine serum (FBS, Gibco, Thermo Fisher Scientific, Waltham, Massachusetts, USA) and 1% penicillin-streptomycin (Biosharp, Labgic Technology, Anhui, China).

The standard conditions for rBMSC cell incubation were set at 37 °C in a humidified atmosphere containing 5% CO_2_. Culture media were replaced every 72 h. Subcultures were performed once 90% cell confluence had been reached. Passage 3 rBMSCs were used for this study. Cryopreservation was conducted by thawing in 1 mL cell cryopreservation solution (Furong Biology, FH7001, Shandong, China) at −80 °C.

### Experimental assays

#### Structural and chemical characterization: scanning electron microscopy (SEM), energy dispersive spectroscopy (EDS), X-ray photoelectron spectroscopy (XPS) and Fourier Transform Infrared-spectroscopy (FTIR)

A morphological analysis of the scaffold surfaces was performed using SEM (VEGA3, TESCAN, Brno, Czech Republic) equipped with energy dispersive spectroscopy (EDS) for elemental distribution and content analysis, along with X-ray photoelectron spectroscopy (XPS, AXIS UltraDLD, Shimadzu). Elemental distribution via EDS was only assessed for PCL-BG-EPL scaffolds. For SEM, all samples were sputtered with Pt prior to analysis for 30 s.

The chemical structure of PCL, PCL-BG, PCL-BG-EPL scaffolds and the BG alone were analyzed using Fourier transform infrared spectroscopy (FTIR, 500–3500 cm^−1^, Thermo Nicolet 6700, Thermo Fisher Scientific, Waltham, Massachusetts, USA).

#### In vitro degradation: weight loss

The degradation resistance of PCL, PCL-BG, and PCL-BG-EPL scaffolds was tested by incubation in Tris-HCl buffer solution (0.1 M, pH 7.4, Sangon Biotech, Shanghai, China). The specimens were weighed (*W*_*0*_) and soaked in 50 mL of Tris-HCl, then incubated at 37 °C for 1, 4, and 8 weeks. At each interval, the specimens were recovered, rinsed three times with PBS, and dried at room temperature overnight before measuring the final weight (*W*_*t*_). To determine the degradation ratio, the following formula was used: (W_0_ – W_t_) / W_0_ × 100. The pH of the Tris-HCl solution was measured with a pH meter at the initial time and each subsequent time point.

#### Antibacterial capacity

A *Staphylococcus aureus* (ATCC 25923) strain was used to assess antibacterial activity using a disk diffusion method [34]. The bacterial strain was cultured on blood agar plates (Nutrition Agar Plate, BKMAM Biotechnology, Hunan, China) at 37 °C in air for 24 h.

After incubation, isolated bacterial colonies from the stock culture were suspended in sterile saline until reaching a turbidity of 0.5 McFarland. A 100 µL sample of *S. aureus* suspension was spread onto the blood agar plate. The PCL and PCL-BG scaffolds served as negative controls, while PCL-BG-EPL scaffolds previously submerged in PBS for 7 [PCL-BG-EPL(7)] and 14 [PCL-BG-EPL(14)] days, as well as scaffolds not submerged before [PCL-BG-EPL(0)], were used as test samples. All scaffolds were placed onto the agar plate, maintaining a 2 cm distance between disks and from the plate edge, and incubated for 18 h at 37 °C. The assay was performed in triplicate, and the results were reported as the average of the independent experimental replicates.

#### Cell viability: confocal laser scanning microscopy (CLSM)

Cell viability was measured using CLSM (Leica Microsystems, Wetzlar, Germany) and the Calcein/PI Cell Viability/Cytotoxicity Assay Kit (Beyotime Biotechnology, Shanghai, China). An initial density of 2 × 10^4^ cells per well was seeded onto the scaffolds in 48-well plates and incubated in a humidified atmosphere at 5% CO_2_ and 37 °C for 3 days.

The scaffolds were washed with PBS to reduce esterase activity, and 1 µL of calcein AM (1000×) and 1 µL of propidium iodide (PI) solutions were added to 1 mL of testing buffer. The scaffolds were then transferred to 35 mm confocal dishes, and 150 µL of solution was added directly to each well and incubated for 30 min at 37 °C before visualization. Confocal laser scanning microscopy was performed using a FITC filter for calcein AM (excitation/emission 494/517 nm) and a RHOD filter for PI (excitation/emission 535/617).

#### Cell proliferation: water-soluble tetrazolium salt (WST-8) assay

A WST-8 assay kit (Enhanced Cell Counting Kit-8, CCK-8, Beyotime Biotechnology, Shanghai, China) was used to examine the proliferation of rBMSC on the scaffolds.

First, an initial density of 2 × 10^4^ cells/well was seeded onto the scaffolds and incubated in a humid atmosphere with 5% CO_2_ at 37 °C for 1, 3 and 7 days. After each study interval, the scaffolds were transferred into new wells containing 500 µL of culture media.

The assay was performed by adding 50 µL of WST-8 reagent and incubating under the same conditions for 2 h. Cell proliferation was measured using a microplate spectrophotometer (Multiskan Go, 1.01.12, Thermo Fisher Scientific, Waltham, Massachusetts, USA) at a test wavelength of 440 nm (A_440_) and reference wavelength of 650 nm (A650).

Cell proliferation was calculated as (A_440_ - A_650_) minus A_blank_, where A_blank_ indicates the absorbance from wells with scaffolds and culture media but without seeded cells. The average value from all biological replicates was recorded as the representative data point.

#### Cytotoxicity: WST-8 assay

A direct cytotoxicity assay was conducted to evaluate the toxic effects of the scaffolds over 7 days. Cells were seeded at a density of 6 × 10^4^ cells/well and cultured in the presence of the test materials. Cytotoxicity was assessed by measuring the reduction in metabolic activity using a WST-8 assay (Enhanced Cell Counting Kit-8, CCK-8, Beyotime Biotechnology, Shanghai, China) according to the protocol previously described (see *cell proliferation* assay).

#### Osteogenic gene expression: reverse transcription-quantitative polymerase chain reaction (RT-qPCR)

To assess the expression of osteogenic-related genes such as alkaline phosphatase (*Alpl*), osteocalcin (*Ocn*), osteopontin (*Opn*), collagen type I (*Col1a1*), and runt-related transcription factor 2 (*Runx2*), a reverse transcription-quantitative PCR (RT-qPCR) was performed. Cells were seeded onto the scaffolds at an initial density of 2 × 10^4^ cells/well, and RT-qPCR was conducted after 1 and 7 days of incubation.

The total RNA was extracted using an RNA purification kit (EZ-press RNA Purification Kit, EZBioscience, Minnesota, USA) following the manufacturer’s protocol. RNA quality and quantity were assessed with spectrophotometry (NanoDrop, Thermo Fisher Scientific, Waltham, Massachusetts, USA) to ensure a ratio of 260/230 ≥ 1.7.

800 ng of total RNA was used for reverse transcription with the Hifair® V one-step RT-gDNA digestion SuperMix for qPCR (Yeasen Biotechnology, Shanghai, China) according to the manufacturer’s protocol.

The qPCR analysis was performed using SYBR Green marker (Hieff® qPCR SYBR Green Master Mix (Low Rox Plus), Yeasen Biotechnology, Shanghai, China). Relative gene expression was calculated using the and normalized to the reference gene Glyceraldehyde 3-phosphate dehydrogenase (*Gapdh*). All primers used for PCR amplification are listed in Table S1 (Sangon Biotech, Shanghai, China).

#### Osteogenic protein synthesis: Western blot

The relative expression levels of ALPL, OCN, OPN, and COL1A1 were measured by performing Western blot on cell lysates. The rBMSC cells were seeded at an initial density of 2 × 10⁴ cells per well in 48-well plates. For each experimental time point, culture media were extracted, and the scaffolds were washed twice with PBS to eliminate serum proteins. Protein extraction was performed using 200 µL of Radio-Immunoprecipitation Assay buffer (RIPA, Beyotime Biotechnology, P0013B, Shanghai, China), containing 1% protease and phosphatase inhibitor cocktail (100×) (Thermo Fisher Scientific, Waltham, Massachusetts, USA) and 1% phenylmethylsulfonyl fluoride (PMSF, Beyotime Biotechnology, P0286, Shanghai, China), followed by up-and-down pipetting. The buffer was transferred into Eppendorf tubes and incubated at 4 °C for 30 min. The resulting protein was mixed with Laemmli buffer (Beyotime Biotechnology, Shanghai, China) in a 1:4 ratio and boiled at 99 °C, 800 rpm for 16 min before being stored at −80 °C.

For each sample, 20 µg of protein were electrophoresed on a 4–20% gradient precast gel (SuperPAGE^TM^, Bis-Tris, 4–20%, EpiZyme Biomedical Technology). Electrophoresed gels were transferred onto polyvinylidene fluoride membranes (PDVF, Immobilon®-P PVDF Membrane, Millipore, Massachusetts, USA). The membranes were blocked in Tris-buffered saline containing Tween-20 with 5% non-fat milk at room temperature for 1 h and incubated with mouse and goat anti-rat primary antibodies (Proteintech Group, Illinois, USA) for ALPL, OCN, OPN, COL1A1, and GAPDH (as the loading control) overnight at 4 °C. Subsequently, goat anti-Mouse and anti-Rabbit secondary antibodies (Multi-rAb, Proteintech Group, Illinois, USA) were incubated at a 1:5000 dilution at room temperature for 1 h, and protein immunoreactivity was detected using enhanced chemiluminescence (ECL, PicoLight, EpiZyme Biomedical Technology).

The intensity of the bands was measured using Image J software (version 1.54p, National Institutes of Health, MD, USA). Gray values were quantified and normalized to the loading control, GAPDH, and the data were expressed as fold-change relative to the PCL control group.

### Data analysis

#### Outcome variables

Qualitative assessments of the results obtained by SEM, FTIR, and XPS was provided for the physical characterization of the scaffolds. Quantitative data was collected for the degradation assay (measured as % of weight change), cell proliferation and cytotoxicity assays (measured in absorbance units (AU)), antibacterial capacity assay (measured in mm of inhibition zone diameter), gene expression assay (measured as relative gene expression using the 2^-ΔΔCt^ method), and protein synthesis assay (measured in fold-change relative protein synthesis).

#### Statistical analysis

Normality was checked using the Shapiro-Wilk test, along with an evaluation of distribution parameters (skewness and kurtosis) and visual inspection of boxplots. All data are reported as mean and standard deviation (SD).

For comparative analyses, one-way ANOVA was used to examine differences between scaffold and control groups across various incubation periods for results obtained from the cell proliferation, cytotoxicity, relative gene expression, and protein synthesis assays. Based on the results of variance homogeneity, post-hoc comparisons were conducted using either Bonferroni’s test or Dunnett’s multiple comparisons test, depending on the homogeneity of variances. A significance level of *p* < 0.05 was applied to all statistical tests. All analyses were performed using IBM SPSS Statistics software (version 29.0.1.1, IBM Corporation, NY, USA).

## Results

### Structural and chemical characterization: scanning electron microscopy (SEM), energy dispersive spectroscopy (EDS), X-ray photoelectron spectroscopy (XPS) and Fourier Transform Infrared-spectroscopy (FT-IR)

**Fig. 1:**
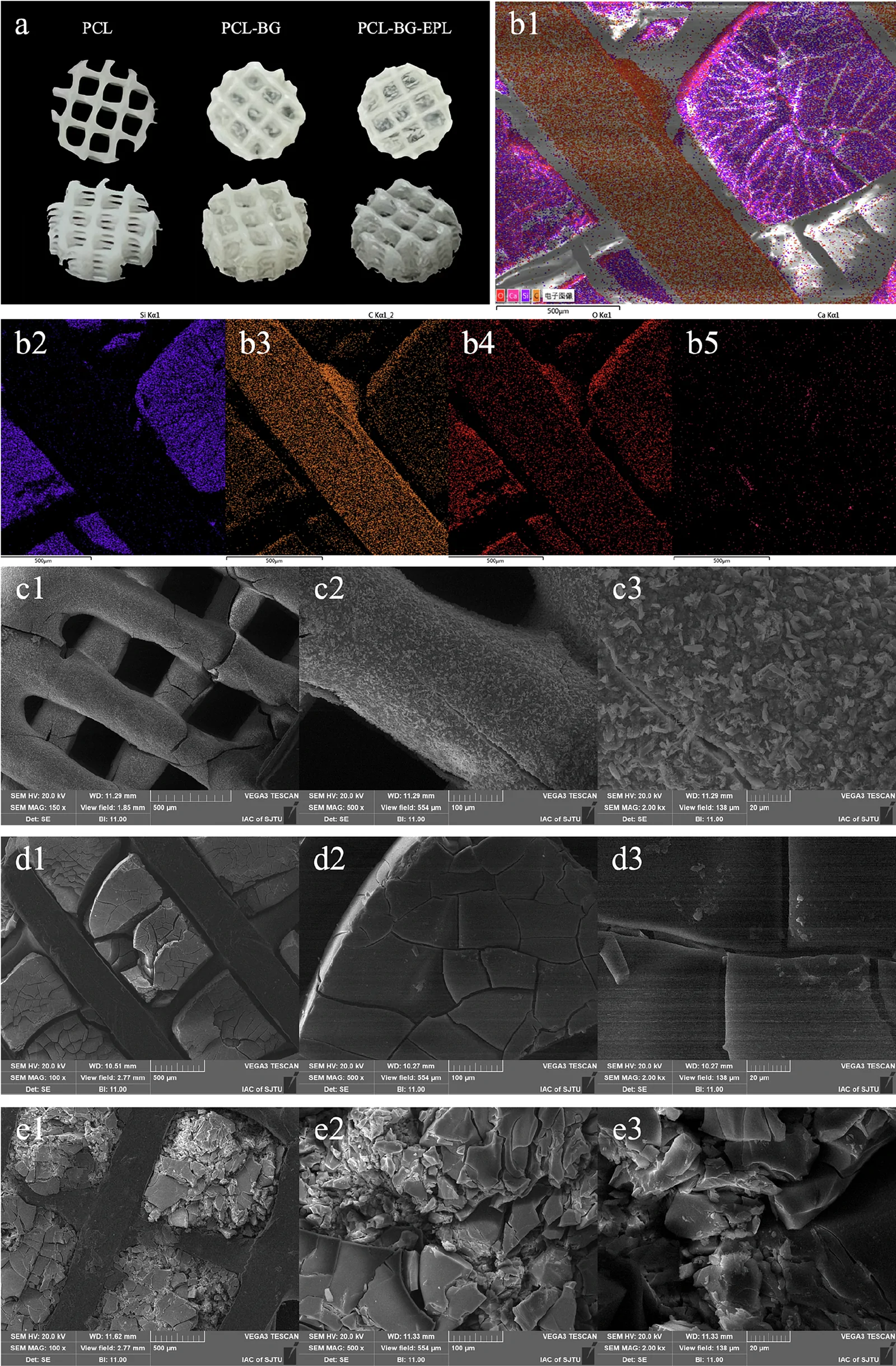
Structural characterization of PCL, PCL-BG, and PCL-BG-EPL scaffolds. **a** Scaffold design, cylindrical shape (5 mm diameter × 3 mm height). **b1** EDS surface element mapping of PCL-BG-EPL scaffolds for elements Si (**b2**), C (**b3**), O (**b4**), and Ca (**b5**). **c1–c3** SEM images of pure PCL scaffolds. **d1–d3** SEM images of PCL-BG scaffolds. **e1–e3** SEM images of PCL-BG-EPL scaffolds

The scaffold microarchitecture was examined using SEM (Fig. 1c). The SEM images showed the surface morphology of PCL, PCL-BG, and PCL-BG-EPL scaffolds, along with how BG was distributed within the polymer matrix. All scaffolds displayed a macroporous structure with pore sizes between 500–700 µm.

The pure PCL scaffolds displayed evenly spaced layers of biomaterial, with a surface topography characterized by micro-fissures and particulate features that increased surface roughness. The BG coating in PCL-BG and PCL-BG-EPL scaffolds showed a homogeneous distribution across the interconnected macropores. While PCL-BG scaffolds appeared smoother, flatter, and more agglomerated, the addition of EPL seemed to change the surface morphology, resulting in a crystalline topography with a rougher surface. The BG agglomerates also showed micro-fissures, further adding to the textured surface of the scaffold. The EDS results demonstrated that Si, C, O, and Ca were evenly distributed within the scaffolds, indicating a uniform coating of BG over the PCL scaffolds and showing the main elements in PCL-BG-EPL scaffolds even after EPL doping (Fig. 1b).

**Fig. 2:**
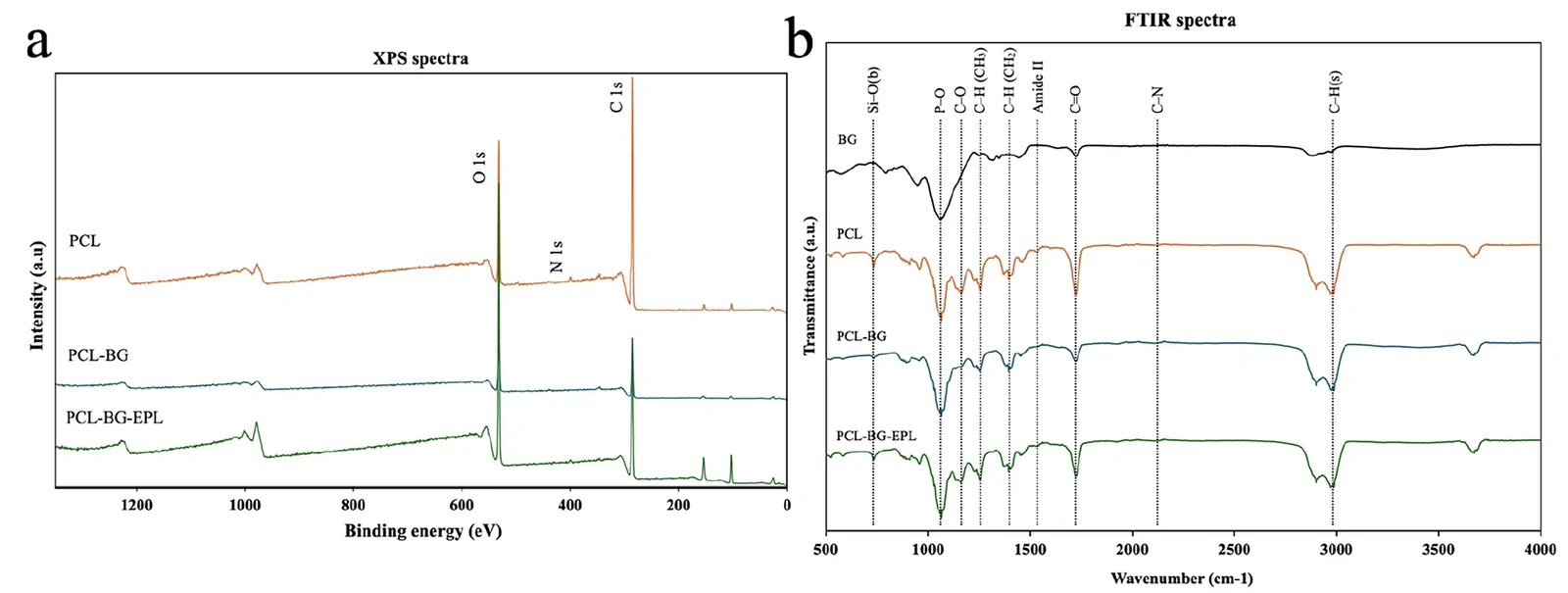
**a** XPS spectra of PCL, PCL-BG, and PCL-BG-EPL scaffolds. **b** FTIR spectra of BG, PCL, PCL-BG, and PCL-BG-EPL scaffolds

X-ray photoelectron spectroscopy (Fig. 2a) was used to analyze the elemental distribution of the scaffolds, confirming the sequential modification of PCL with BG and EPL. Pure PCL showed characteristic C 1 s (286 eV) and O 1 s (533 eV) peaks corresponding to its ester-containing backbone, with no detectable nitrogen signal. After BG incorporation, the O 1 s peak intensity increased, and additional contributions from inorganic elements became more evident, consistent with the presence of Si–O–Si and Si–O–Ca bonds. Following EPL functionalization, a small N 1 s (400 eV) peak appeared, demonstrating the introduction of nitrogen-containing groups on the composite surface. These findings confirm the successful incorporation of BG and subsequent EPL modification of the PCL matrix.

Complementary FTIR analysis (Fig. 2b) of the scaffolds further confirmed the presence of characteristic functional group vibrations, verifying the successful integration of BG and EPL into the polymer matrix. The spectrum of pure BG exhibited a dominant band at 1040 cm^−1^ corresponding to Si-O-Si asymmetric stretching vibrations [35]. Pure PCL scaffolds exhibited strong transmittance peaks at 2970 and 2900 cm^−1^ (corresponding to asymmetric and symmetric C-H stretching, respectively), 1723 cm^−1^ (ester C = O stretching), and 1160 cm^−1^ (C-O-C stretching) [36]. After adding BG, the spectra displayed new, more intense peaks in the 1000 to 1100 cm^−1^ range, indicating P-O stretching, along with a band at 820 cm^−1^ corresponding to Si-O bending, which confirms the presence of silica groups from the BG [37]. Following EPL loading, additional broad absorption appeared between 1500 and 1650 cm^−1^ (amide II), along with subtle shifts near the ester C = O band, suggesting hydrogen bonding interactions between EPL amines and PCL carbonyl groups [38]. Overall, these complementary XPS and FTIR results validate the successful fabrication and surface functionalization of the PCL-BG-EPL composite scaffold.

### In vitro degradation: weight loss

The mean weight for PCL scaffolds at the initial timepoint was 12.37 mg (SD = 0.79), PCL-BG scaffolds averaged 34.04 mg (SD = 3.26), and PCL-BG-EPL scaffolds averaged 33.80 mg (SD = 2.38). The in vitro degradation of all scaffold types, submerged in a Tris-HCl solution, resulted in significant weight loss over an 8-week experimental period. PCL scaffolds averaged weight losses of 12.12% (SD = 1.15), while PCL-BG and PCL-BG-EPL scaffolds reached weight losses of up to 28.12% (SD = 2.08) and 25.71% (SD = 1.17), respectively, after 8 weeks of soaking. PCL-BG and PCL-BG-EPL scaffolds underwent a more significant weight loss as compared to the PCL controls (*p* < 0.001). However, there were no statistically significant differences in weight loss between the two BG-coated scaffolds at any of the study intervals (Table 1).

**Table 1:** In vitro degradation test after soaking in Tris-HCl solution for 1, 4, and 8 weeks

In vitro degradation test								
Scaffold type	N	Initial weight [mg (SD)]	Weight loss [% (SD)]			p value (between time points)		
			1 W	4 W	8 W	1 W vs 4 W	4 W vs 8 W	1 W vs 8 W
PCL	9	12.37 (0.79)	3.16 (1.20)	5.00 (0.73)	12.12 (1.15)	0.045	0.014	0.003
PCL-BG	9	34.04 (3.26)	19.93 (3.50)	27.80 (2.09)	28.12 (2.08)	0.002	1.000	0.001
PCL-BG-EPL	9	33.80 (2.38)	19.62 (2.05)	25.07 (2.01)	25.71 (1.17)	0.001	1.000	<0.001
p value (between scaffolds)	PCL vs PCL-BG	<0.001	<0.001	<0.001				
	PCL vs PCL-BG-EPL	<0.001	<0.001	<0.001				
	PCL-BG vs PCL-BG-EPL	1.000	0.272	0.217				

### Antibacterial capacity

The antibacterial capacity of PCL-BG-EPL scaffolds was tested against *S. aureus* using the disk diffusion method (Table 2). The scaffolds showed varying antibacterial effects depending on their soaking time in PBS. Non-soaked scaffolds had an average inhibition zone diameter of 11.00 mm (SD = 1.73). In contrast, PCL-BG-EPL(7) (soaked for 7 days) scaffolds exhibited reduced antibacterial activity, with inhibition zones averaging 8.33 mm (SD = 0.58). PCL-BG-EPL(14) (soaked for 14 days) scaffolds, along with the negative controls (PCL-BG and PCL scaffolds), showed no antibacterial activity, as no inhibition zones were observed in any of the assay replicates.

**Table 2:** Antibacterial activity against *S. aureus* during a disk diffusion test

Antibacterial capacity						
Bacteria	N	Inhibition zone diameter [mm (SD)]				
		PCL	PCL-BG	PCL-BG-EPL(0)	PCL-BG-EPL(7)	PCL-BG-EPL(14)
*S. aureus*	3	0 (0)	0 (0)	11.00 (1.73)	8.33 (0.58)	0 (0)

### Cell viability: confocal laser scanning microscopy (CLSM)

**Fig. 3:**
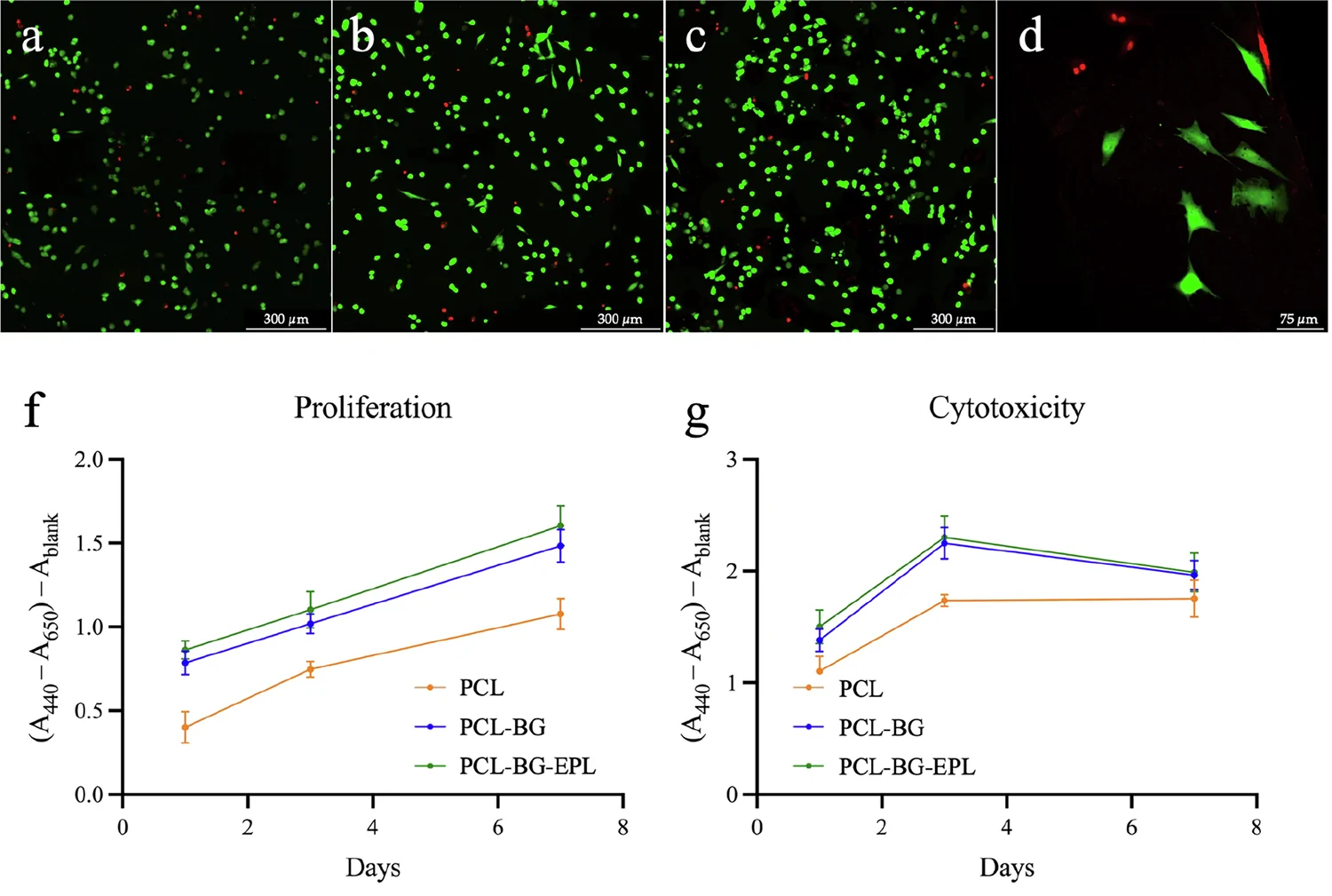
Cell viability, proliferation and cytotoxicity of rBMSCs in each scaffold group. Representative Live/Dead staining images after 3 days of incubation of each scaffold group **a** PCL, **b** PCL-BG, **c** PCL-BG-EPL, **d** PCL-BG-EPL (200×). **f** Cell proliferation profiles after 1, 3, and 7 days of incubation. **g** Cytotoxicity profiles after 1, 3 and 7 days of incubation

Confocal microscopy evaluation showed that rBMSCs attached and formed a uniform distribution across all scaffold surfaces. This attachment was confirmed by high viability ratios observed through Live/Dead staining, demonstrating that all three scaffold types supported a metabolically active cell population (Fig. 3a–d). Magnified images displayed viable cells with a spindle-shaped morphology and multiple cytoplasmic extensions.

### Cell proliferation: water-soluble tetrazolium salt (WST-8) assay

Cell proliferation of rBMSCs growing on all three scaffold groups significantly increased with longer incubation times (*p* < 0.001). The comparison between scaffold groups showed increased cell proliferation on PCL-BG and PCL-BG-EPL scaffolds compared to the pure PCL controls (*p* < 0.001); however, there were no statistically significant differences when comparing PCL-BG to PCL-BG-EPL scaffolds at any of the study intervals (Fig. 3f, Table S2).

### Cytotoxicity: WST-8 assay

The direct cytotoxicity assay showed a significant increase in optical density for all scaffold groups during the first 3 days of incubation. After this period, pure PCL scaffolds maintained a similar absorbance up to day 7 of incubation [1.73 AU (SD = 0.05) vs 1.75 AU (SD = 0.18)], while PCL-BG and PCL-BG-EPL scaffolds exhibited a significant decrease in cell metabolic activity when comparing both incubation times [PCL-BG: 2.25 AU (SD = 0.14) vs 1.96 AU (SD = 0.13), *p* = 0.007; PCL-BG-EPL: 2.30 AU (SD = 0.19) vs 1.99 AU (SD = 0.17), *p* = 0.002]. When comparing the biological performance of the scaffolds between time points, both test groups performed significantly better than control scaffolds after 1 and 3 days of incubation; however, at the 7-day mark, no differences in absorbance were observed among any of the scaffold groups (Fig. 3g, Table S3).

### Osteogenic gene expression: reverse transcription-quantitative polymerase chain reaction (RT-qPCR)

All scaffolds actively promoted osteogenic differentiation of rBMSCs, as evidenced by the increased expression of key osteogenic marker genes: *Alpl*, *Ocn*, *Opn*, *Col1a1*, and *Runx2* (Table S4).

The gene expression detected for rBMSCs growing on PCL scaffolds was used as a reference to assess the fold change in gene expression of PCL-BG and PCL-BG-EPL scaffolds after 1 and 7 days of incubation.

**Fig. 4:**
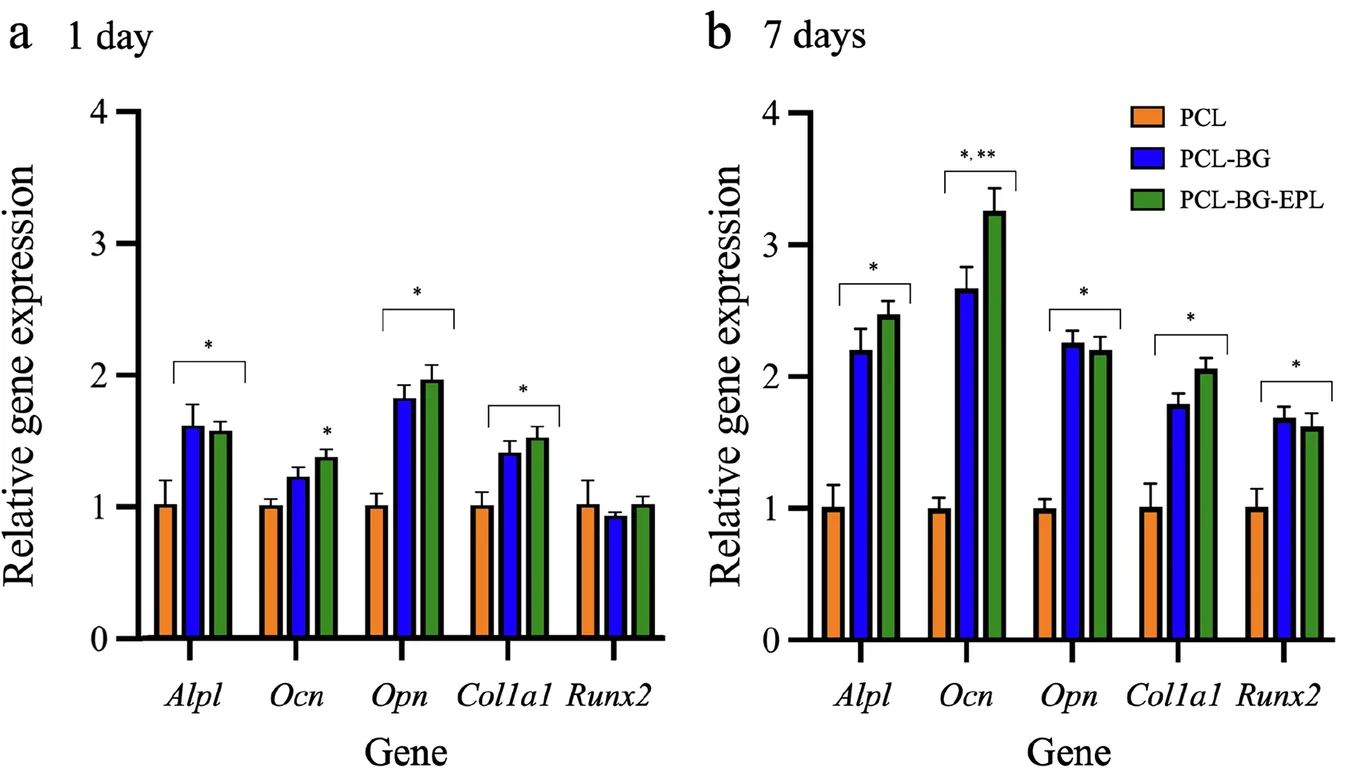
Relative gene expression profiles of osteogenic markers (*Alpl*, *Ocn*, *Opn*, *Col1a1*, and *Runx2*) in rBMSCs co-cultured on PCL, PCL-BG, and PCL-BG-EPL scaffolds, normalized to *Gapdh*, and analyzed by RT-qPCR at 1 day (**a**) and 7 days (**b**). PCL scaffolds are used as reference group. (*Alpl*: alkaline phosphatase; *Ocn*: osteocalcin; *Opn*: osteopontin; *Col1a1*: type 1 collagen; *Runx2*: runt-related transcription factor 2). Asterisks mark significant differences (* significantly different from PCL control; ** significant difference between PCL-BG and PCL-BG-EPL scaffolds)

After 1 day (Fig. 4a), rBMSCs on PCL-BG scaffolds showed significantly higher expression of osteogenic genes compared to those on PCL scaffolds, with *Alpl*, *Opn*, and *Col1a1* levels increasing by 1.61-, 1.82-, and 1.40-fold, respectively (*p* < 0.05). The PCL-BG-EPL scaffolds also exhibited a significant upregulation of *Ocn* (1.37-fold) at this time point (*p* < 0.05). By day 7 (Fig. 4b), both experimental groups demonstrated significant upregulation of all targeted genes compared to the control (*p* < 0.001).

However, when comparing both BG-coated scaffolds with and without EPL-loading, there were no differences in gene expression for any of the target genes at any study intervals.

### Osteogenic protein synthesis: western blot

**Fig. 5:**
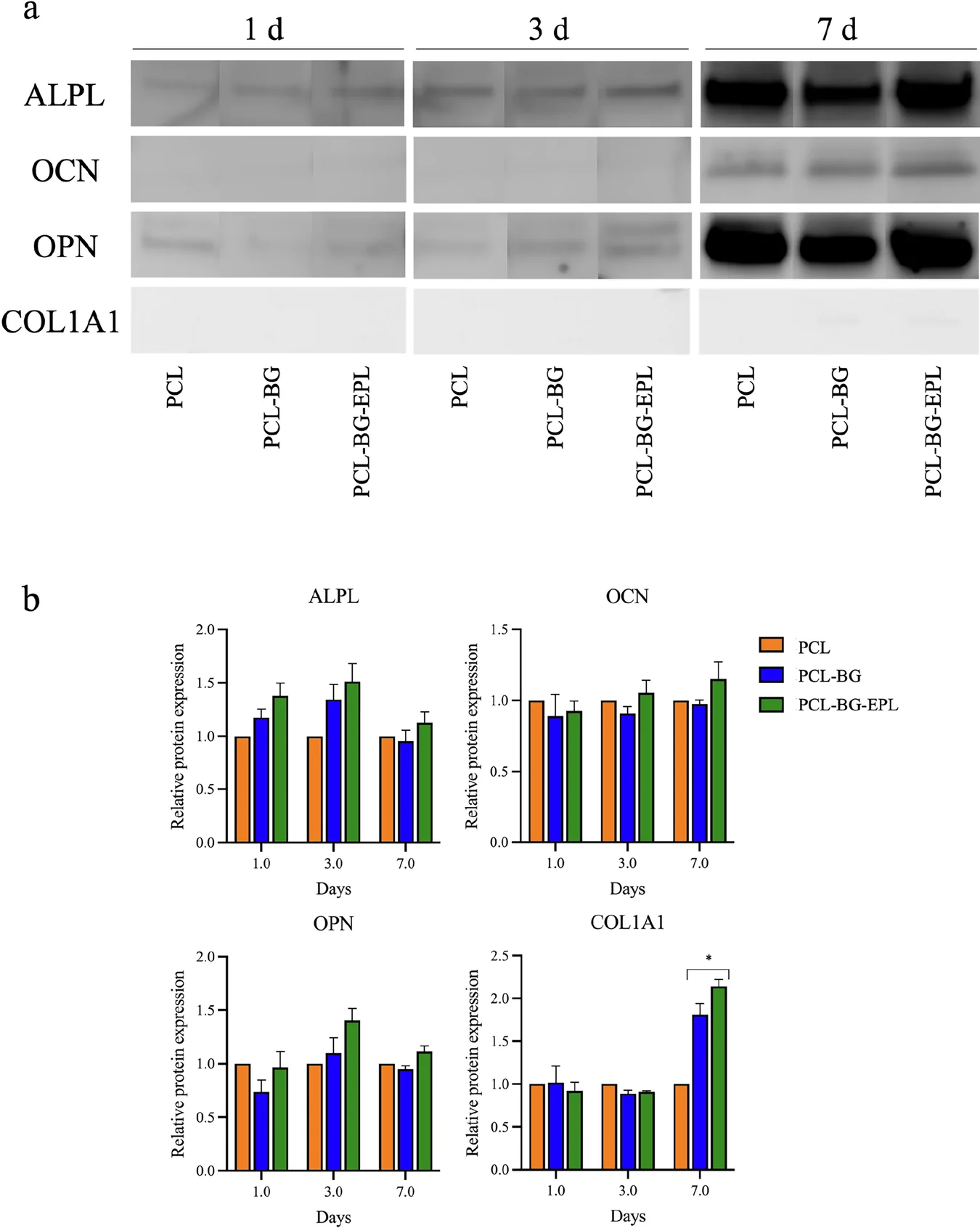
Relative protein expression of osteogenic markers (ALPL, OCN, OPN, COL1A1) in rBMSCs co-cultured on PCL, PCL-BG, and PCL-BG-EPL scaffolds, normalized to GAPDH, and analyzed by RT-qPCR over 7 days: **a** western blots for the different target proteins; **b** Comparison of fold-changes in protein synthesis using PCL scaffolds as reference (ALPL: alkaline phosphatase; OCN: osteocalcin; OPN: osteopontin; COL1A1: type 1 collagen). Asterisks mark significant differences (* significantly different from PCL control)

To verify that the observed upregulation of osteogenic genes led to increased production of functional proteins, protein expression was analyzed using western blot. Whole cell lysates were prepared to measure the intracellular levels of osteogenic proteins (ALPL, OCN, OPN, and COL1A1). The immunoassay confirmed the production of ALPL, OCN, and OPN by rBMSCs cocultured with all three scaffold groups; however, COL1A1 was only detected in samples from PCL-BG and PCL-BG-EPL scaffolds on day 7 (Fig. 5).

Band width and intensity for all target proteins increased with longer incubation times. However, compared to the PCL scaffold group, the statistical analysis showed no significant differences in relative protein synthesis among the three scaffold groups, except for COL1A1, which had significantly higher synthesis at the 7-day incubation time on PCL-BG-EPL scaffolds. Nonetheless, the band intensity for this protein remained weak (Table S5).

## Discussion

This in vitro study evaluated the structural and biological properties of 3D-printed, BG-coated, and EPL-loaded PCL scaffolds, with a focus on their potential applications in bone tissue engineering. In this context, 3D printing and additive manufacturing have emerged as promising technologies to offer point-of-care, customized alternatives to traditional bone replacement grafts [39]. The coating with BG provided several benefits to the polymeric base, including enhanced bioactivity and the ability to facilitate sustained-release drug delivery [23, 40]. The in vivo performance of surface modifications with bioactive glass-coated implants demonstrated an increased percentage of new bone formation around coated compared to non-coated materials after 15 and 30 days of implantation [41].

The surface change observed after EPL-loading of BG-coated scaffolds, transitioning from smoother, agglomerated structures in PCL-BG to a distinct crystalline topography in PCL-BG-EPL, can be attributed to the process of EPL functionalization. In this process, EPL molecules, a polycationic polymer, likely interact with the negatively charged BG surface through electrostatic interactions and hydrogen bonding [29]. The subsequent soaking in distilled water probably causes a partial dissolution and reprecipitation of the glass phase or the formation of apatite crystalline aggregates on the glass surface [35, 37]. The uniform distribution of the BG coating, observed in both SEM and EDS mapping, was a key finding, indicating successful coating that creates a continuous bioactive interface across the entire scaffold rather than isolated patches. This uniformity is crucial for predictable bioactivity, ion release, and the formation of the apatite layer. The EDS mapping confirmed the presence of Si and Ca elements across the scaffold surface. Although the EDS analysis was performed only for the PCL-BG-EPL scaffolds, it can be reasonably assumed that the same considerations apply to PCL-BG scaffolds.

The chemical analyses provided by XPS and FTIR offer evidence of the step-by-step modification of the scaffold. The XPS spectra show the appearance of a nitrogen signal (N 1 s) after EPL functionalization, which is absent in the PCL-BG spectrum, serving as a direct indicator of EPL conjugation. The increase in oxygen and silane signals after BG coating aligns with the coverage of the inorganic BG layer onto the polymer matrix. The FTIR results complement the XPS data by identifying specific molecular bonds and functional groups introduced at each stage. In FTIR spectra from both PCL-BG and PCL-BG-EPL scaffolds, the presence of silanol (Si–OH) and phosphate (P–O) groups confirms the presence of bioactive glasses [35, 42], indicating interactions between the PCL ester groups and the surface silanol groups of BG. After EPL functionalization, the appearance of amide II bands and the changes observed in the ester carbonyl (C = O) region suggest hydrogen bonding between the amine groups from EPL and the carbonyl groups on the BG surface [38].

This non-covalent interaction, while providing a stable attachment for EPL, represents a potential limitation of the current design. The relatively low surface area and pore volume of conventional BG restrict the amount of EPL that can be adsorbed and may lead to premature release or reduced long-term antimicrobial efficacy [43]. In contrast, the incorporation of mesoporous bioactive glasses (MBG), with its highly ordered pore structure and significantly larger specific surface area, could enhance EPL loading efficiency, binding stability, and controlled release behavior, offering a promising direction for future scaffold optimization [23, 44].

In determining the in vitro degradation kinetics of PCL scaffolds and composite PCL-based scaffolds, it has been observed that mass loss is significantly affected by the construct architecture [45]. The results obtained in this experiment are consistent with those reported in other studies using BG-coated scaffolds, showing weight losses of up to 67% after 12 weeks of immersion in PBS and approximately 40% at 8 weeks [18]. The degradation process of BGs itself has also been shown to positively affect bone cell proliferation and differentiation, as the constituents of BG —namely, Si, Ca, and P —have been proven to stimulate genes in osteoblastic cells [46].

The integration of EPL into BG-PCL scaffolds provides antibacterial activity, effectively preventing bacterial adhesion and biofilm formation. This function may be vital in avoiding bacterial infections after surgery in clinical settings. The proposed mechanism involves EPL disrupting microbial cell wall integrity and cell membrane permeability [47, 48]. Local delivery of this antimicrobial peptide (AMP) offers several advantages for bone regeneration: (1) it bypasses the limited blood flow in surgical sites, overcoming the reduced effectiveness of systemic antibiotics [49]; (2) in light of the rising global burden of antimicrobial resistance, antimicrobial peptides like EPL are promising broad-spectrum options that can help reduce the development of resistance [27, 50, 51]. Although clinical use of AMPs faces hurdles like poor pharmacokinetics, toxicity, and breakdown by proteases [52], our results show that EPL loaded onto BG-PCL scaffolds maintains its antimicrobial activity against *S. aureus for* up to 7 days after soaking in PBS. This suggests a protective role against common pathogens in bone infections. However, the antimicrobial activity should be reassessed under physiological conditions for a better understanding of the pharmacokinetics of PCL-BG-EPL scaffolds.

The cell proliferation assay showed sustained attachment and growth of rBMSCs over 7 days on both BG-coated scaffolds, with no significant difference in proliferation patterns between them. However, the pure PCL scaffolds exhibited significantly lower cell proliferation at all time points compared to the test scaffolds. This is attributed to high hydrophobicity, which results in lower cell adhesion and consequently lower absorbance readings throughout the incubation periods. Previous studies have shown that bioactive glass-coated scaffolds promote cell attachment and adhesion by increasing surface roughness, as demonstrated by white light interferometry [53].

Regarding cytotoxicity, a significant increase in cytotoxicity was detected for both test scaffolds when comparing day 3 and day 7 of incubation. This is probably due to the static nature of the in vitro assays, were the BG is slowly dissolved into the media [46], leading to a rapid ion saturation and reprecipitation over the scaffolds. Although there are concerns about cytotoxic effects of AMPs [52, 54], this report found no significant differences in cell proliferation or cytotoxicity between EPL-loaded and non-loaded PCL-BG scaffolds, indicating that the slow release of EPL does not reach cytotoxic levels when incorporated onto the BG surface.

It is widely accepted that bioactive glasses, especially Bioglass® 45S5 composition (by weight: 45% SiO_2_, 24.5% Na_2_O, 24.4% CaO, and 6% P_2_O_5_), can upregulate osteogenic gene expression [55]. Consistent with this, our results showed that rBMSCs co-cultured with both PCL-BG and PCL-BG-EPL scaffolds exhibited a significant increase in *Alpl*, *Ocn*, *Opn*, *Col1a1*, and *Runx2 expression* compared to those on pure PCL scaffolds. This clear genetic profile indicates that the BG-coating notably enhanced the differentiation of rBMSCs toward an osteogenic lineage. Importantly, the presence of EPL did not reduce this effect, as the PCL-BG-EPL scaffolds performed similarly to their non-EPL-loaded counterparts, suggesting that its antimicrobial properties can be added without compromising bioactivity.

While RT-qPCR analysis showed a clear upregulation of osteogenic gene expression, western blot analysis did not demonstrate a directly proportional increase in protein levels at the same time points. This difference can be explained by the complexity of gene regulation, including variations in protein turnover rates or a time lag between transcription and translation. Therefore, we hypothesize that the initial pro-osteogenic stimulus provided by the dissolving BG coating likely triggers a rapid transcriptional response, preparing for matrix production, with the subsequent translation and secretion of mature proteins occurring at a later stage [56]. Nonetheless, we conclude that the osteogenic effect is mainly due to the BG component, not the EPL-loading.

This series of in vitro assays collectively forms the basis for using 3D-printed PCL-BG-EPL scaffolds in bone tissue engineering. However, several limitations must be highlighted. First, the results come from a controlled monoculture environment, which does not reflect the complexity of an in vivo setting, including immune response, a dynamic vascular system, and the interaction of multiple cell types during bone healing. Future work should validate these findings in critical-sized bone defect models in vivo to evaluate scaffold performance, biocompatibility, immune response, and bone regeneration in a physiologically relevant environment. Regarding antimicrobial activity, while the efficacy against *S. aureus* was demonstrated for at least 7 days, the release profile and long-term stability were not fully measured, and testing against a single bacterial species does not fully represent the challenge of multispecies biofilms. Future research should expand antimicrobial testing to multispecies biofilm models and include detailed pharmacokinetic studies of EPL release. Furthermore, replacing conventional BGs with mesoporous bioactive glass (MBG) as the bio ceramic coating may enhance the scaffold’s drug-loading capacity, EPL retention stability, and controlled release kinetics [23].

## Conclusions

In this study, PCL, PCL-BG, and PCL-BG-EPL scaffolds were successfully created using fused filament fabrication, BG dip-coating, and EPL-loading. The results demonstrated that these scaffolds had good technical properties and enhanced biological capabilities compared to pure PCL scaffolds. Functionalization with EPL showed increased antibacterial properties compared to PCL-BG or pure PCL scaffolds. No major differences in biological performance were found between PCL-BG and PCL-BG-EPL scaffolds, except for their antibacterial activity. This proof-of-concept study confirms that the PCL-BG-EPL scaffold is a promising candidate for further in vivo investigation as a bone graft substitute, combining the osteogenic properties of BG with the antibacterial functionality of EPL.

## Supplementary information

Supplementary information
