# Supplementary material for: Enhanced osteogenesis and antibacterial activity of bioactive glass-coated, epsilon-poly-L-lysine-loaded, polycaprolactone 3D-printed scaffolds with potential use in bone tissue engineering
Source: J Mater Sci Mater Med. 2026 May 5;37(1):133. doi: 10.1007/s10856-026-07065-8 (PMC13724660; doi:10.1007/s10856-026-07065-8)
Supplement: Supplementary file 1 [file 10856_2026_7065_MOESM1_ESM.docx]

**Table S1.** qPCR primers for the target (*Alpl*, *Ocn*, *Opn*, *Col1a1*, and *Rux2*) and housekeeping (*Gapdh*) genes.

| Gene | Primer | Sequence |
| --- | --- | --- |
| *Gapdh* | Forward | 5’-GATGGTGAAGGTCGGTGTG-3’ |
|  | Reverse | 5’-ATGAAGGGGTCGTTGATGG-3’ |
| *Opn* | Forward | 5’-CGAGGTGATATAGTGTGGTTTATGG-3’ |
|  | Reverse | 5’-GCACCATTCAACTCCTCGCTTTC-3’ |
| *Ocn* | Forward | 5’-CGCTACCTGTATCAATGGCTGG-3’ |
|  | Reverse | 5’-CTCCTGAAAGCCGATGTGGTCA-3’ |
| *Col1a1* | Forward | 5’- CACTGCAAGAACAGCGTAGC-3’ |
|  | Reverse | 5’-AAGTTCCGGTGTGACTCGTG-3’ |
| *Alpl* | Forward | 5’-AGATGGATGAGGCCATCGGA-3’ |
|  | Reverse | 5’-CCAAACGTGAAAACGTGGGA-3’ |
| *Runx2* | Forward | 5’-CAGACCAGCAGCACTCCATA-3’ |
|  | Reverse | 5’-AGACTCATCCATTCTGCCGC-3’ |

**Table S2.** Cell proliferation of PCL, PCL-BG, and PCL-BG-EPL scaffolds measured by spectrophotometry using a CCK-8 assay, measured at 1, 3, and 7 days.

| Cell proliferation | | | | | | | | | |
| --- | --- | --- | --- | --- | --- | --- | --- | --- | --- |
| **Scaffold type** | **N** | | **Optical density [AU (SD)]** | | | **p value**  **(between time points)** | | |  |
|  |  |  | 1 d | 3 d | 7 d | 1 d vs  3 d | 3 d vs  7 d | 1 d vs  7 d |  |
| **PCL** | 8 | | 0.40 (0.09) | 0.74 (0.05) | 1.08 (0.09) | <0.001 | <0.001 | <0.001 |  |
|  |  |  |  |  |  |  |  |  |  |
| **PCL-BG** | 8 | | 0.78 (0.07) | 1.02 (0.06) | 1.48 (0.10) | <0.001 | <0.001 | <0.001 |  |
|  |  |  |  |  |  |  |  |  |  |
| **PCL-BG-EPL** | 8 | | 0.86 (0.05) | 1.10 (0.11) | 1.60 (0.12) | <0.001 | <0.001 | <0.001 |  |
|  |  |  |  |  |  |  |  |  |  |
| **p value (between scaffolds)** | PCL vs PCL-BG | | <0.001 | <0.001 | <0.001 |  |  |  |  |
|  | PCL vs PCL-BG-EPL | | <0.001 | <0.001 | <0.001 |  |  |  |  |
|  | PCL-BG vs PCL-BG-EPL | | 1.000 | 1.000 | 0.235 |  |  |  |  |

**Table S3.** Direct cytotoxicity of rBMSCs seeded onto PCL, PCL-BG, and PCL-BG-EPL scaffolds measured by spectrophotometry using a CCK8 assay, measured at 1, 3, and 7 days.

| Cytotoxicity | | | | | | | | |
| --- | --- | --- | --- | --- | --- | --- | --- | --- |
| **Scaffold type** | **N** | | **Optical density [AU (SD)]** | | | **p value**  **(between time points)** | | |
|  |  |  | 1 d | 3 d | 7 d | 1 d vs 3 d | 3 d vs 7 d | 1 d vs 7 d |
| **PCL** | 8 | | 1.10 (0.13) | 1.73 (0.05) | 1.75 (0.18) | <0.001 | 1.000 | <0.001 |
|  |  |  |  |  |  |  |  |  |
| **PCL-BG** | 8 | | 1.38 (0.10) | 2.25 (0.14) | 1.96 (0.13) | <0.001 | 0.007 | <0.001 |
|  |  |  |  |  |  |  |  |  |
| **PCL-BG-EPL** | 8 | | 1.50 (0.15) | 2.30 (0.19) | 1.99 (0.17) | <0.001 | 0.002 | <0.001 |
|  |  |  |  |  |  |  |  |  |
| **p value (between scaffolds)** | PCL vs PCL-BG | | 0.009 | <0.001 | 0.260 |  |  |  |
|  | PCL vs PCL-BG-EPL | | <0.001 | <0.001 | 0.099 |  |  |  |
|  | PCL-BG vs PCL-BG-EPL | | 1.000 | 1.000 | 1.000 |  |  |  |

**Table S4.** Gene expression fold-changes in rBMSCs co-cultured on PCL-BG, and PCL-BG-EPL scaffolds relative to cells on PCL scaffolds using the 2^−ΔΔ^*^Ct^* method.

| Gene expression | | | | | | |
| --- | --- | --- | --- | --- | --- | --- |
| **Gene** | **Infill** | **N** | | **Relative gene expression**  **[Mean (SD)]** | | **p value** |
|  |  |  |  | 1 d | 7 d |  |
| ***Alpl*** | **PCL** | 3 | | 1.01 (0.18) | 1.01 (0.17) | 1.000 |
|  |  |  |  |  |  |  |
|  | **PCL-BG** | 3 | | 1.61 (0.16) | 2.20 (0.17) | 0.005 |
|  |  |  |  |  |  |  |
|  | **PCL-BG-EPL** | 3 | | 1.57 (0.07) | 2.47 (0.10) | <0.001 |
|  |  |  |  |  |  |  |
|  | **p value (between scaffolds)** | PCL vs PCL-BG | | 0.004 | <0.001 |  |
|  |  | PCL vs PCL-BG-EPL | | 0.008 | <0.001 |  |
|  |  | PCL-BG vs PCL-BG-EPL | | 1.000 | 0.647 |  |
| ***Ocn*** | **PCL** | 3 | | 1.00 (0.05) | 1.00 (0.07) | 1.000 |
|  |  |  |  |  |  |  |
|  | **PCL-BG** | 3 | | 1.22 (0.07) | 2.67 (0.16) | <0.001 |
|  |  |  |  |  |  |  |
|  | **PCL-BG-EPL** | 3 | | 1.37 (0.06) | 3.26 (0.17) | <0.001 |
|  |  |  |  |  |  |  |
|  | **p value (between scaffolds)** | PCL vs PCL-BG | | 0.499 | <0.001 |  |
|  |  | PCL vs PCL-BG-EPL | | 0.022 | <0.001 |  |
|  |  | PCL-BG vs PCL-BG-EPL | | 1.000 | <0.001 |  |
| ***Opn*** | **PCL** | 3 | | 1.00 (0.09) | 1.00 (0.06) | 1.000 |
|  |  |  |  |  |  |  |
|  | **PCL-BG** | 3 | | 1.82 (0.10) | 2.26 (0.09) | 0.001 |
|  |  |  |  |  |  |  |
|  | **PCL-BG-EPL** | 3 | | 1.96 (0.11) | 2.20 (0.10) | 0.131 |
|  |  |  |  |  |  |  |
|  | **p value (between scaffolds)** | PCL vs PCL-BG | | <0.001 | <0.001 |  |
|  |  | PCL vs PCL-BG-EPL | | <0.001 | <0.001 |  |
|  |  | PCL-BG vs PCL-BG-EPL | | 1.000 | 1.000 |  |
| ***Col1a1*** | **PCL** | 3 | | 1.00 (0.10) | 1.01 (0.18) | 1.000 |
|  |  |  |  |  |  |  |
|  | **PCL-BG** | 3 | | 1.40 (0.09) | 1.79 ((0.08) | 0.012 |
|  |  |  |  |  |  |  |
|  | **PCL-BG-EPL** | 3 | | 1.52 (0.08) | 2.06 (0.08) | <0.001 |
|  |  |  |  |  |  |  |
|  | **p value (between scaffolds)** | PCL vs PCL-BG | | 0.012 | <0.001 |  |
|  |  | PCL vs PCL-BG-EPL | | 0.001 | <0.001 |  |
|  |  | PCL-BG vs PCL-BG-EPL | | 1.000 | 0.164 |  |
| ***Runx2*** | **PCL** | 3 | | 1.01 (0.18) | 1.00 (0.14) | 1.000 |
|  |  |  |  |  |  |  |
|  | **PCL-BG** | 3 | | 0.92 (0.03) | 1.69 (0.08) | <0.001 |
|  |  |  |  |  |  |  |
|  | **PCL-BG-EPL** | 3 | | 1.01 (0.06) | 1.62 (0.10) | <0.001 |
|  |  |  |  |  |  |  |
|  | **p value (between scaffolds)** | PCL vs PCL-BG | | 1.000 | <0.001 |  |
|  |  | PCL vs PCL-BG-EPL | | 1.000 | <0.001 |  |
|  |  | PCL-BG vs PCL-BG-EPL | | 1.000 | 1.000 |  |

**Table S5.** Relative protein expression of ALPL, OCN, OPN, COL1A1 in rBMSCs seeded onto PCL-BG, and PCL-BG-EPL scaffolds, represented as fold-change relative to expression levels of cells seeded on PCL scaffolds.

| Protein synthesis | | | | | | | | | |
| --- | --- | --- | --- | --- | --- | --- | --- | --- | --- |
| **Gene** | **Infill** | **N** | | **Relative protein synthesis [Mean (SD)]** | | | **p value**  **(between time points)** | | |
|  |  |  |  | 1 d | 3 d | 7 d | 1 d vs 3 d | 3 d vs 7 d | 1 d vs 7 d |
| **ALPL** | **PCL** | 3 | | 1.00 (0.00) | 1.00 (0.00) | 1.00 (0.00) | 1.000 | 1.000 | 1.000 |
|  |  |  |  |  |  |  |  |  |  |
|  | **PCL-BG** | 3 | | 1.18 (0.08) | 1.34 (0.14) | 0.96 (0.10) | 0.798 | 0.127 | 0.362 |
|  |  |  |  |  |  |  |  |  |  |
|  | **PCL-BG-EPL** | 3 | | 1.37 (0.12) | 1.51 (0.17) | 1.13 (0.10) | 0.983 | 0.316 | 0.448 |
|  |  |  |  |  |  |  |  |  |  |
|  | **p value (between scaffolds)** | PCL vs PCL-BG | | 0.313 | 0.266 | 0.313 |  |  |  |
|  |  | PCL vs PCL-BG-EPL | | 0.170 | 0.194 | 0.170 |  |  |  |
|  |  | PCL-BG vs PCL-BG-EPL | | 0.559 | 0.964 | 0.697 |  |  |  |
|  |  |  |  |  |  |  |  |  |  |
| **OCN** | **PCL** | 3 | | 1.00 (0.00) | 1.00 (0.00) | 1.00 (0.00) | 1.000 | 1.000 | 1.000 |
|  |  |  |  |  |  |  |  |  |  |
|  | **PCL-BG** | 3 | | 0.89 (0.15) | 0.91 (0.05) | 0.97 (0.03) | 1.000 | 0.710 | 0.993 |
|  |  |  |  |  |  |  |  |  |  |
|  | **PCL-BG-EPL** | 3 | | 0.93 (0.07) | 1.05 (0.09) | 1.15 (0.12) | 0.760 | 0.990 | 0.478 |
|  |  |  |  |  |  |  |  |  |  |
|  | **p value (between scaffolds)** | PCL vs PCL-BG | | 0.952 | 0.411 | 0.822 |  |  |  |
|  |  | PCL vs PCL-BG-EPL | | 0.803 | 0.984 | 0.706 |  |  |  |
|  |  | PCL-BG vs PCL-BG-EPL | | 1.000 | 0.552 | 0.607 |  |  |  |
|  |  |  |  |  |  |  |  |  |  |
| **OPN** | **PCL** | 3 | | 1.00 (0.00) | 1.00 (0.00) | 1.00 (0.00) | 1.000 | 1.000 | 1.000 |
|  |  |  |  |  |  |  |  |  |  |
|  | **PCL-BG** | 3 | | 0.74 (0.11) | 1.10 (0.13) | 0.95 (0.03) | 0.250 | 0.786 | 0.418 |
|  |  |  |  |  |  |  |  |  |  |
|  | **PCL-BG-EPL** | 3 | | 0.96 (0.15) | 1.40 (0.11) | 1.11 (0.05) | 0.179 | 0.234 | 0.861 |
|  |  |  |  |  |  |  |  |  |  |
|  | **p value (between scaffolds)** | PCL vs PCL-BG | | 0.301 | 0.952 | 0.471 |  |  |  |
|  |  | PCL vs PCL-BG-EPL | | 1.000 | 0.141 | 0.345 |  |  |  |
|  |  | PCL-BG vs PCL-BG-EPL | | 0.703 | 0.383 | 0.152 |  |  |  |
|  |  |  |  |  |  |  |  |  |  |
| **COL1A1** | **PCL** | 3 | | 1.00 (0.00) | 1.00 (0.00) | 1.00 (0.00) | 1.000 | 1.000 | 1.000 |
|  |  |  |  |  |  |  |  |  |  |
|  | **PCL-BG** | 3 | | 1.02 (0.19) | 0.89 (0.04) | 1.81 (0.13) | 0.975 | 0.022 | 0.062 |
|  |  |  |  |  |  |  |  |  |  |
|  | **PCL-BG-EPL** | 3 | | 0.92 (0.10) | 0.91 (0.07) | 2.14 (0.09) | 1.000 | 0.008 | 0.001 |
|  |  |  |  |  |  |  |  |  |  |
|  | **p value (between scaffolds)** | PCL vs PCL-BG | | 1.000 | 0.242 | 0.046 |  |  |  |
|  |  | PCL vs PCL-BG-EPL | | 0.928 | 0.290 | 0.011 |  |  |  |
|  |  | PCL-BG vs PCL-BG-EPL | | 1.000 | 0.996 | 0.231 |  |  |  |
